# Chemotherapy-Induced Oxidative Stress in Pediatric Acute Lymphoblastic Leukemia

**DOI:** 10.7759/cureus.35968

**Published:** 2023-03-10

**Authors:** Preety Chaudhary, Sweta Kumari, Pooja Dewan, Sunil Gomber, Rafat S Ahmed, Mrinalini Kotru

**Affiliations:** 1 Pediatrics and Child Health, University College of Medical Sciences, Delhi, IND; 2 Pediatrics, University College of Medical Sciences, Delhi, IND; 3 Pediatrics/Oncology, University College of Medical Sciences, Delhi, IND; 4 Biochemistry, University College of Medical Sciences, Delhi, IND; 5 Pathology, University College of Medical Sciences, Delhi, IND

**Keywords:** oxidative stress, leukemia, chemotherapy, childhood cancer, antioxidant

## Abstract

Introduction

Plasma antioxidant capacity in children receiving chemotherapy decreases due to the effect of the disease and chemotherapy. Increased oxidative stress (OS) predisposes to an increased risk for chemotherapy-related toxicity and febrile neutropenic episodes.

Materials and methods

We conducted this case-control study in the hematology-oncology unit of the department of pediatrics of a tertiary hospital in Delhi, India, from November 2017 to March 2019 to compare OS between children with acute lymphoblastic leukemia (ALL) and healthy controls. We estimated the trends in OS as measured by the plasma total antioxidant capacity (TAC) and thiobarbituric acid reactive substance (TBARS) levels at baseline and at the completion of induction I (four weeks), induction II (eight weeks), and induction IIA-consolidation (16 weeks) phases of chemotherapy in children with ALL. We also assessed the change in OS during different phases of initial treatment and studied the association between OS and the hematological toxicity of chemotherapy (determined by the need for blood component therapy and the number of febrile neutropenic episodes) and serum cobalamin and folate levels.

Results

OS was significantly higher in children with ALL at diagnosis (n=23) compared to controls (n=19). The median (interquartile range (IQR)) TAC levels (mM) were significantly lower (1.21 (1.05-1.26) versus 1.28 (1.26-1.32), P=0.006), and TBARS levels (nmol/mL) were significantly higher (312.0 (216.6-398.0) versus 58.5 (46.2-67.2), P<0.001) in children with ALL at diagnosis compared to controls. OS was highest at the end of the induction I phase (four weeks) despite the patients being in clinical and hematological remission. OS at the completion of intensive chemotherapy (16 weeks) was higher than at diagnosis. A significant correlation was found between serum folate levels and TAC levels at baseline (P=0.03). Serum cobalamin levels, the need for blood component therapy, and the number of febrile neutropenic episodes did not have any association with OS.

Conclusion

Children with ALL had significantly higher OS compared to controls, indicating that underlying disease affects the oxidative balance unfavorably. Chemotherapy itself increases oxidative stress.

## Introduction

Acute lymphoblastic leukemia (ALL) accounts for more than 50% of hematopoietic malignancies in childhood [1]. In low- and middle-income countries, chemotherapy remains the mainstay of treatment in children with ALL. It has been shown that in ALL, both the underlying neoplasm and chemotherapy contribute to increased oxidative stress (OS), which is defined as an imbalance between increased levels of reactive oxygen species (ROS) and a low activity of antioxidant mechanisms [2,3].

The normal balance between the production of ROS and antioxidant activity maintains the body in equilibrium and allows normal cell differentiation. However, in leukemia, this balance is shifted toward excessive production of ROS and a decrease in antioxidant activity due to the hypermetabolic state of the neoplastic cells. Increased OS in the bone marrow milieu leads to DNA damage and mutagenesis potentiating leukemogenesis [4]. Several studies have shown that underlying leukemia itself contributes to the increased OS as evidenced by low antioxidant levels in children with ALL at initial diagnosis [5-11]. Interestingly, chemotherapy has also been shown to produce oxidative damage, which leads to apoptosis of cancerous cells and therefore aids therapeutic effect [12]. Unfortunately, the free radicals generated by chemotherapeutic drugs and radiation not only destroy tumor cells but also normal cells, leading to toxic side effects such as neurotoxicity, hematological toxicity, and mucositis [13-15]. The increased ROS adversely affects hematopoietic stem cells and accounts for the myelotoxic side effects of chemotherapy drugs [16,17].

Thus, the dual effects of OS in leukemogenesis and chemotherapeutic effects are perplexing. It is still unclear if the increased OS should be prevented given that OS plays a role in leukemogenesis as well as chemotherapeutic side effects [12]. Suppressing this OS may in fact be counterproductive given that some chemotherapeutic agents work by increasing the OS. Understanding the dynamics of antioxidant levels and their relationship with chemotherapeutic toxicity may help foster future opportunities to develop therapeutic strategies for countering oxidative damage by chemotherapeutic drugs without compromising on chemosensitivity in children with ALL [18]. While oxidant damage generates lipid peroxidation products such as malondialdehyde (MDA), which are measured in the form of thiobarbituric acid reactive substances (TBARS) in plasma, total antioxidant capacity (TAC) is an integrated parameter that estimates the cumulative action of all antioxidants present in plasma and body fluids. Thus, the measurement of TAC and TBARS can help understand the overall OS in the body.

We conducted this study with the primary objective of comparing plasma OS as measured by TAC and TBARS levels in children with ALL compared to healthy controls. The secondary objective was to assess the change in OS during different phases of initial treatment and evaluate the association between OS at diagnosis and hematological toxicity of chemotherapy (measured by the need for blood component therapy and the number of febrile neutropenic episodes during the initial treatment), and serum cobalamin and folate levels.

## Materials and methods

This case-control study was conducted from November 2017 to March 2019 in the pediatric hematology-oncology unit of the department of pediatrics of a tertiary hospital in Delhi, India. Written informed consent was taken from the caregivers, and ethical clearance was obtained from the Institutional Ethics Committee for Human Research of the University College of Medical Sciences, Delhi (reference number: IECHR/2017/32/89). Based on the study by Mazor et al. [10], where the TAC levels in children with ALL were estimated as 1.43 (± 0.1) mM/L compared to 1.25 (± 0.2) mM/L in healthy controls, a sample size of 19 children per group was calculated at 80% power and alpha error of 0.05, using an online sample size calculator [19]. Based on our previous records, we expected a 20% mortality rate in children with ALL during the initial four weeks of induction treatment; therefore, a sample size of 23 children with newly diagnosed ALL and 19 controls was needed to compare the TAC levels between the two groups.

A diagnosis of ALL was established based on morphological evaluation, cytochemistry, and immunophenotyping of bone marrow aspirate and biopsy specimens according to the criteria described by the World Health Organization (WHO) [20]. Age-matched 19 healthy children were enrolled as controls. Controls were selected from among the healthy siblings of the patients with ALL and from the healthy children visiting the pediatric outpatient department for routine immunization. Children with ALL who had already been started on treatment or were receiving any alternative/complementary therapy for cancer including supplementation with antioxidants, children presenting with relapsed ALL, and those suffering from chronic illnesses such as diabetes and asthma were excluded.

All children with ALL were treated using the modified MCP 841 protocol developed jointly by India and the National Cancer Institute (NCI), Bethesda, USA [21]; the protocol is less intense with manageable toxicity of chemotherapy and hence considered more suited to low- and middle-income countries. This protocol includes an initial 16 weeks of intensive chemotherapy followed by maintenance chemotherapy over the next 18 months.

We estimated the OS using the levels of TAC and TBARS, a lipid peroxidation by-product, in the plasma of participants. Raised TBARS and low TAC levels were regarded as increased OS. Plasma TAC was measured using Cayman’s kit (Cayman Chemicals, Ann Arbor, MI, USA), which relies on the ability of antioxidants in the sample to inhibit the oxidation of 2,2’-azino-di-(3-ethylbenzthiazoline sulfonate) (ABTS) by metmyoglobin. The amount of ABTS produced can be monitored by reading the absorbance at 405 nm. The capacity of the antioxidants in the sample to prevent ABTS oxidation is compared with that of Trolox, a water-soluble tocopherol analog, and is quantified as millimolar Trolox equivalents. Plasma TBARS was measured using the method of Satoh [22], which is based on the principle that in the presence of heat and acid, the MDA present in the plasma reacts with thiobarbituric acid to produce a colored end product that absorbs light at 532 nm. The intensity of color developed corresponds to the level of lipid peroxidation, i.e., TBARS levels in the plasma sample.

TAC and TBARS were measured at the time of enrolment, post-induction I phase (four weeks), post-induction II phase (eight weeks), and induction IIA-consolidation (16 weeks) phase of chemotherapy. In the control group, TAC and TBARS were estimated only at the time of enrolment. Serum cobalamin and serum folate levels were estimated by electro-chemiluminescence using Cobas e411 automated analyzer at the time of enrolment in both cases and controls. Detailed clinical evaluation and relevant investigations were done as per the institutional protocol at diagnosis and as indicated during the treatment. All children were observed for chemotherapy-induced toxicity as per common toxicity criteria [23], the number of febrile neutropenic episodes, and disease progression/remission.

Statistical analysis

Data were analyzed using the Statistical Package for the Social Sciences (SPSS) software version 25 (IBM SPSS Statistics, Armonk, NY, USA). Considering the small sample size, we used nonparametric tests for statistical analysis. Baseline characteristics and outcome measures on continuous scales were expressed as median (interquartile range (IQR)), and comparisons between groups were made using the Mann-Whitney U test. Wilcoxon signed-rank test was used to compare the sequential values of TAC and TBARS during different phases of treatment. The Spearman correlation coefficient was used for determining the correlation between antioxidant status and chemotherapy-related toxic outcomes, outcomes of induction therapy, serum folate levels, serum cobalamin levels, and the number of febrile neutropenic episodes during treatment. Statistical significance was established if the P value was less than 0.05.

## Results

**Figure 1:**
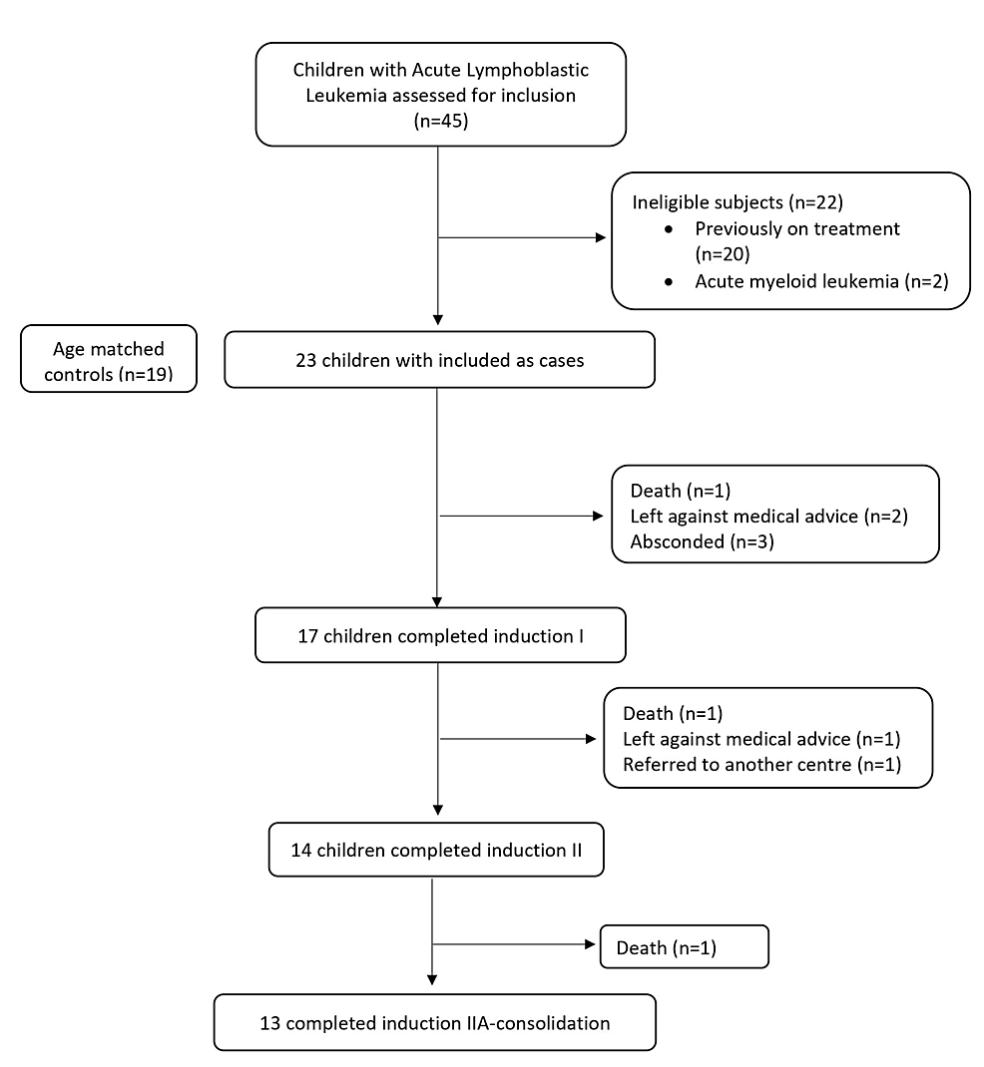
Flow of participants in the study

We recruited 23 newly diagnosed children with ALL as the case group and 19 children as the control group. All children with ALL were diagnosed as B-cell ALL based on the flow cytometric profile. The median age (months) of children with ALL and healthy controls was 60 (36-122) and 72 (48-108), respectively (P=0.53). Among 23 children included with newly diagnosed B-ALL, 13 were males. Twelve out of 19 participants in the control group were males. A total of 17 cases completed induction I (initial four weeks of therapy), 14 cases completed induction II (eight weeks of therapy), and 13 cases completed induction IIA-consolidation (16 weeks of therapy). Figure 1 outlines the screening, recruitment, and outcome of participants in the study.

**Figure 2:**
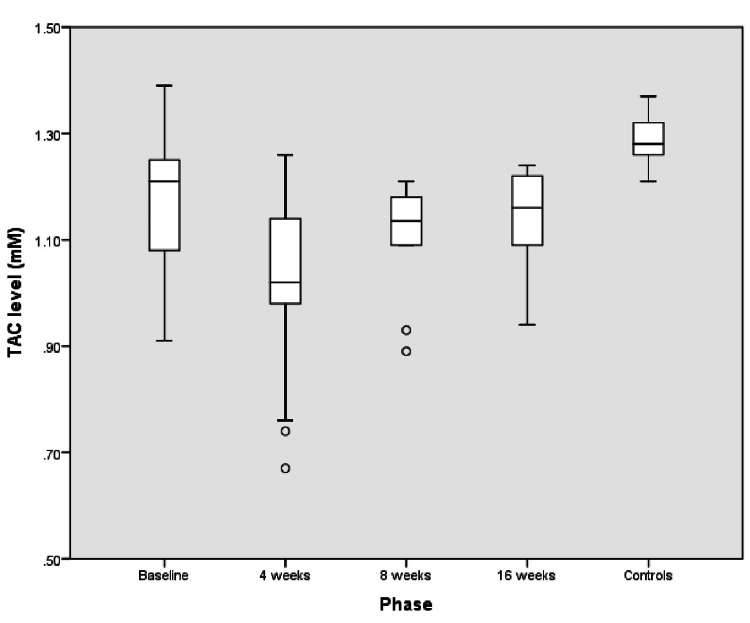
TAC (mM) levels in controls and children with ALL at baseline and at the end of induction I (four weeks), induction II (eight weeks), and induction IIA-consolidation (16 weeks) phases of treatment TAC: total antioxidant capacity, ALL: acute lymphoblastic leukemia

**Figure 3:**
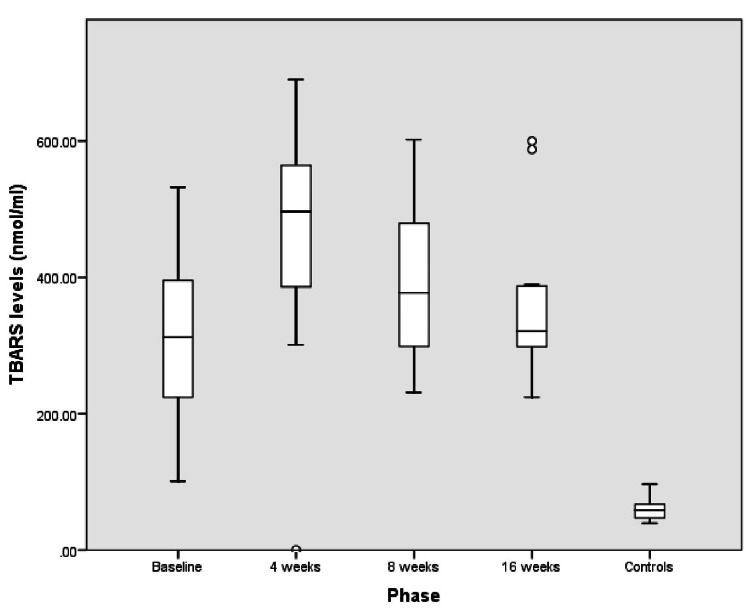
TBAR (nmol/mL) levels in controls and children with ALL at baseline and at the end of induction I (four weeks), induction II (eight weeks), and induction IIA-consolidation (16 weeks) phases of treatment TBARS: thiobarbituric acid reactive substances, ALL: acute lymphoblastic leukemia

Children with ALL at diagnosis had much higher OS compared to the control population; the median (interquartile range (IQR)) TAC levels (mM) were significantly lower (1.21 (1.05-1.26) versus 1.28 (1.26-1.32), P = 0.006), and TBARS levels (nmol/mL) were significantly higher (312 (216.6-398) versus 58.5 (46.2-67.2), P<0.001) in children with ALL at diagnosis compared to controls (Figure 2 and Figure 3).

The OS was highest at the end of the induction I phase of chemotherapy (four weeks) in children with ALL, as depicted in Table 1.

**Table 1:** Comparison of oxidative stress in controls and children with ALL at baseline and at the end of induction I, induction II, and induction IIA-consolidation phases of treatment *Values expressed as median (IQR) ALL: acute lymphoblastic leukemia, IQR: interquartile range, TAC: total antioxidant capacity, TBARS: thiobarbituric acid reactive substance

Oxidative markers	Baseline	Post-induction I	Post-induction II	Post-induction IIA-consolidation
TAC (mM)				
Children with ALL	1.21 (1.05-1.26)*	1.02 (0.91-1.13)*	1.13 (1.09-1.18)*	1.16 (1.09-1.22)*
Controls	1.28 (1.26-1.32)*	-	-	-
P value	0.006	-	-	-
TBARS (nmol/mL)				
Children with ALL	312.00 (216.60-398.00)*	496.10 (366.30-580.20)*	377.45 (297.90-502.30)*	321.00 (297.80-388.00)*
Controls	58.50 (46.20-67.20)*	-	-	-
P value	<0.001	-	-	-

The OS at the completion of intensive chemotherapy (induction IIA-consolidation) at the end of 16 weeks was higher than at diagnosis. All surviving children (n=13) were in clinical and hematological remission at 16 weeks of treatment.

We found a significant positive correlation between serum folate levels and TAC levels at initial diagnosis (P=0.03). However, no significant correlation was seen between OS and serum cobalamin levels, the need for blood component therapy, and the total number of febrile neutropenic episodes (Table 2).

**Table 2:** Correlation between oxidative stress and serum cobalamin levels, serum folate levels, number of febrile neutropenic episodes, and the need for blood component therapy in children with ALL r: Spearman correlation coefficient, P: significant value, ALL: acute lymphoblastic leukemia

Parameter	Total antioxidant capacity		Thiobarbituric acid reactive substances	
	Correlation coefficient (r)	P value	Correlation coefficient (r)	P value
Serum cobalamin levels (pg/mL)	-0.33	0.08	-0.114	0.60
Serum folate levels (ng/mL)	0.44	0.03	0.009	0.69
Number of febrile neutropenic episodes	0.08	0.76	-0.28	0.28
Number of blood transfusions	-0.22	0.92	-0.22	0.31
Number of packed red blood cells transfused	0.05	0.8	-0.33	0.12
Number of platelets transfused	-0.80	0.72	-0.13	0.57

## Discussion

Acute lymphoblastic leukemia is a state of hypermetabolism resulting in the excessive generation of free radical species that causes a compromise in the antioxidant defense system and an increased OS [24,25]. We observed that OS (measured in terms of lower TAC levels and raised TBARS levels) was significantly elevated in children with ALL at diagnosis, which is in consonance with previous studies [6,10,26]. Battisti et al. [6] found significantly higher levels of plasma TBARS in patients with ALL at diagnosis as compared to healthy controls, whereas Neyestani et al. [26] reported significantly lower TAC levels in patients with ALL compared to controls. Mazor et al. [10] studied the antioxidant status in terms of thiol plasma levels and the ferric-reducing ability of plasma (FRAP) and found it to be significantly higher in controls compared to children with ALL. This reiterates that the disease process of ALL tilts the oxidative balance unfavorably in children.

We observed that OS increased significantly after chemotherapy was started and reached the peak at the end of induction I (four weeks) despite the bone marrow being in morphological remission. The increased OS may be attributed to the higher tumor burden and intense chemotherapeutic regimens used for the treatment. Similar trends of higher OS in children with ALL at diagnosis and at the end of the induction I phase were reported in previous studies [10,12,27]. Compared to our study, Battisti et al. [6] measured enzymatic antioxidant activity in children with ALL and noted the nadir of antioxidants at the time of disease diagnosis unlike the end of induction as seen in the present study. This difference may be due to the different chemotherapy protocols used and racial and ethnic differences in the study populations.

We reported higher TBARS levels in children with ALL during the initial 4-6 months of chemotherapy compared to controls with the levels at the end of induction IIA-consolidation being higher than at diagnosis. This sustained OS during chemotherapy was also reported by Battisti et al. [6]. Unlike our study, Devi et al. [28] reported normal levels of plasma lipid peroxidation products in untreated ALL patients, which was attributed to the increased activity of enzymatic antioxidants such as glutathione peroxidase and superoxide dismutase. However, we did not assess these enzymatic antioxidants in our study.

The OS in cancer children was highest at the end of induction I and continued to remain high even at the end of induction IIA-consolidation. This finding must be seen in the light that it occurred with a concomitant remission status seen in all 13 survivor children, suggesting that ongoing chemotherapy does not allow the OS to abate to control levels. The persistently high OS in ALL, as evident by lower TAC and increased TBARS during the initial 4-6 months of chemotherapy, may in fact be a marker of chemotherapeutic efficacy as ROS production during chemotherapy leads to cell death and sensitizes tumor cells to cytotoxic chemotherapy. Previously, Naz et al. [29] demonstrated that sustained low TAC was associated with complete remission status in leukemia. The trend for OS during the first 4-6 months of treatment suggests a sustained increase in OS, which should be followed up during the maintenance phase. Thus, we can conclude that the higher OS in ALL is not only a consequence of underlying disease but also due to ongoing chemotherapy.

We noted a significant correlation between serum folate levels and TAC at baseline, which can help explain the therapeutic mechanisms of antifolate drugs such as 6-mercaptopurine used in ALL. No significant correlation was observed between TBARS and serum cobalamin and serum folate levels. We also did not find a significant association between the need for blood component therapy and the number of febrile neutropenic episodes with OS. This may be due to many other factors affecting the bone marrow milieu other than OS.

The major strength of our study is the sequential estimation of OS during different phases of chemotherapy in children with ALL. Previously, most studies have assessed OS at diagnosis alone [9]. Also, adverse events were studied prospectively rather than from case records as has been in previous studies. The study has limitations, which include a small sample size and a lack of data on long-term outcomes.

## Conclusions

Oxidative stress is significantly higher in children with ALL secondary to the underlying disease process or chemotherapeutic drugs. As the treatment progresses, the redox equilibrium worsens despite the remission of the disease, suggesting that chemotherapy acts by oxidative damage to cancer cells. The routine use of antioxidant supplements in these children may be counterproductive as it may hinder the chemotherapeutic action.
